# The microvascular endothelium of the blood-brain barrier is highly restrictive to JC Polyomavirus neuroinvasion

**DOI:** 10.1128/spectrum.00282-25

**Published:** 2025-03-25

**Authors:** Avraham S. Lukacher, Bethany A. O'Hara, Wenqing Yuan, Kaitlin Garabian, Jacob Kaiserman, Evan MacLure, Sheila A. Haley, Walter J. Atwood

**Affiliations:** 1 Department of Cell Biology, Biochemistry, and Molecular Biology, Brown University 6752 , Providence, Rhode Island, USA; University of Manitoba, Winnipeg, Manitoba, Canada

**Keywords:** Polyomavirus, blood-brain barrier, neuroinvasion, PML, JCPyV, extracellular vesicles

## Abstract

JC Polyomavirus is the causative agent of progressive multifocal leukoencephalopathy (PML), an often-fatal demyelinating disease. Unfortunately, a diagnosis of PML occurs only after patients have suffered irreversible neuropathologies. The first step in the initiation of PML is viral entry to the brain, but the route and mechanisms responsible for neuroinvasion have not been well established. To gain a better understanding of this, we asked whether purified virus or virus associated with extracellular vesicles (EVs) could penetrate two different cell culture models of the blood-brain barrier. In one model, we used the hCMEC/D3 brain endothelial cell line, and in the other, we used pluripotent stem cells induced to a brain endothelial cell phenotype (iPSC-EC). We found that neither cell type was permissive to viral infection, but the virus bound and was internalized by both in a sialic acid-dependent manner. Despite virus internalization into these cells, very few virions or virus-associated extracellular vesicles (virus-EVs) penetrated the barriers. The small amount of virus or virus-EVs that did pass through either barrier was sufficient to establish infection in human glial cells. Our findings demonstrate that limited amounts of infectious virions and virus-associated EVs can traverse the brain microvascular endothelium and establish infection.

**IMPORTANCE:**

The human polyomavirus, JC Polyomavirus (JCPyV), causes a rapidly progressing demyelinating disease in immunocompromised or immunomodulated patients. Demyelinating lesions are often seen surrounding blood vessels in the brain. In this paper, we used two models to recapitulate a minimal blood-brain barrier and found that both were highly restrictive of virus penetration. A small amount of virus succeeded in crossing both barriers and was sufficient to establish infection of human glia. These data have direct implications for mechanisms used by JCPyV to invade the CNS and cause neurological disease.

## INTRODUCTION

JC Polyomavirus (JCPyV) is a ubiquitous, double-stranded DNA virus that is the causative agent of progressive multifocal leukoencephalopathy (PML), an often-fatal demyelinating disease (1–4). Approximately 60% of the US population is seropositive for JCPyV, which establishes a persistent, asymptomatic infection in the kidneys (5, 6). JCPyV virions are routinely shed through the urine and stool, and virus is spread via the fecal-oral route (7, 8). In a subset of immunosuppressed individuals, including people with HIV-AIDS and multiple sclerosis patients being treated with immunomodulatory drugs, JCPyV undergoes extensive genomic recombination to a neuropathogenic strain that infects glial cells to cause PML (9–11). A recent study using mouse polyomavirus (MuPyV) as an *in vivo* model for JCPyV pathogenesis showed that neuropathogenic virus quasispecies emerge in the periphery following impaired adaptive immunosurveillance, indicating that neurovirulent JCPyV might originate in the kidneys, escape to peripheral circulation, and enter the brain to cause PML (12).

Once in the brain, lytic infection of glial cells, including astrocytes and oligodendrocytes, results in the formation of demyelinating lesions with a high degree of morbidity and mortality (1–4, 13, 14). JCPyV infection involves viral binding to the α2,6 sialic acid-linked glycan lactoseries tetrasaccharide c (LSTc) receptor motif. JCPyV virions also depend on subsequent interactions with 5-hydroxytryptamine 2 receptors (5-HT2R) to enter cells via clathrin-mediated endocytosis (2, 15–24). Our group has also shown that JCPyV associates with extracellular vesicles (JCPyV-EVs) that can infect cells independently of viral attachment and entry receptors (25, 26). A recent clinical report identified JCPyV archetype DNA associated with EVs in the serum of HIV^+^ patients at risk for PML (27). EVs have been demonstrated to cross the BBB via clathrin-mediated endocytosis, caveolae-mediated endocytosis, and macropinocytosis under different experimental contexts, suggesting that peripheral EVs may serve as a vehicle for JCPyV neuroinvasion (28).

In this report, we investigated JCPyV neuroinvasion using two *in vitro* models of the BBB endothelium: the SV40/hTERT immortalized brain microvascular endothelial line hCMEC/D3 and induced pluripotent stem cell-derived endothelial cells (iPSC-ECs). hCMEC/D3 cells are well-established to represent primary brain microvascular endothelial cells and have been commonly used to model the BBB endothelium *in vitro* (29, 30). In the last decade, iPSC-ECs have been developed to better capture the functionally impermeable properties of the BBB. Similar to a physiological brain microvascular endothelium, iPSC-derived endothelial monolayers exhibit polarization of ABC efflux transporters, high transendothelial electrical resistance (TEER), and low permeability to small molecular weight fluorescent tracers (31).

In this study, we showed that hCMEC/D3 cells and iPSC-ECs are not susceptible to infection but bind JCPyV virions in a sialic acid-dependent manner. Using the transwell barrier system to model the BBB endothelium, we showed that monolayers of hCMEC/D3 cells and iPSC-ECs restrict the penetration of both JCPyV and JCPyV-EVs. However, a small amount of virus or virus-associated EVs successfully crossed both barriers and was sufficient to infect glial cells.

## RESULTS

### hCMEC/D3 cells and iPSC-ECs bind JCPyV and are not susceptible to infection

**Fig 1:**
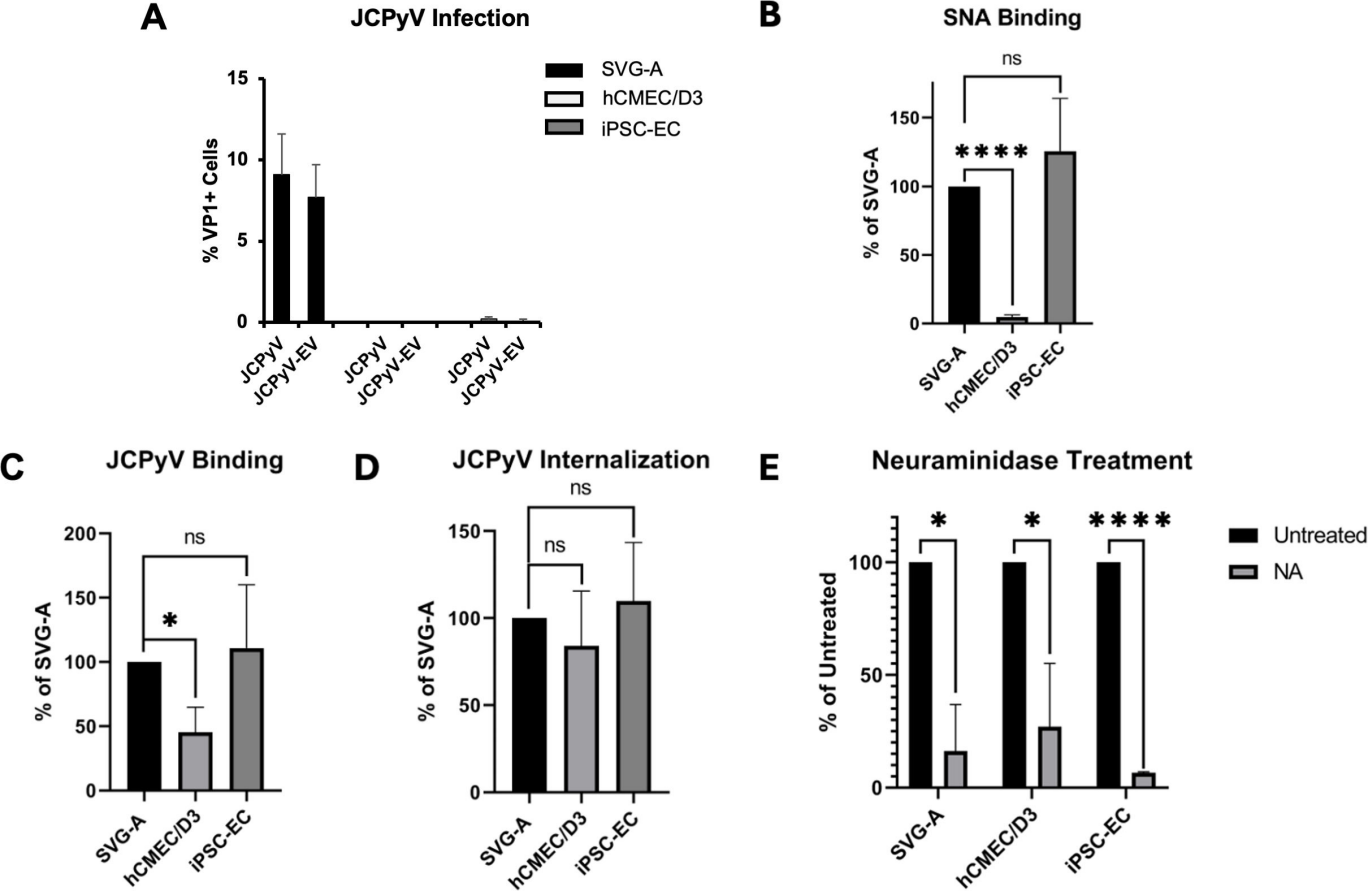
JCPyV interactions with human brain microvascular endothelial cells. (A) hCMEC/D3 and iPSC-EC cells were infected with purified JCPyV and JCPyV-EV, alongside SVG-A controls, and scored for VP1+ cells at 3 dpi. Infection is shown as the percent VP1+ cells vs total cell count. (B) Flow cytometric analysis of α2,6-linked sialic acid expression by SNA binding. (C) Flow cytometric analysis of JCPyV binding. Cells were incubated with Alexa Fluor 488 labeled JCPyV in ice-cold PBS to promote binding and prevent internalization, followed by a trypan blue quench step. Binding data is reported as the total fluorescence minus the post trypan quench (internalized) signal. (D) Flow cytometric analysis of JCPyV internalization. Cells were incubated with Alexa Fluor 488-labeled JCPyV at 37°C, 5% CO_2_ to promote viral internalization, followed by a trypan blue quench step. Internalization data is reported as the post-trypan fluorescent signal. (E) Cells were pretreated with type II neuraminidase (NA) to cleave α2,6-linked sialic acid, and binding of fluorescently-labeled virus was assessed by flow cytometry. For (B–D), fluorescence values were normalized to that of SVG-A cells. For (E), values were normalized to untreated controls. Significance was calculated by a Student’s two-tailed *t* test (ns, nonsignificant, *P < 0.05, ****P < 0.0001).

hCMEC/D3 cells and iPSC-ECs were exposed to JCPyV or JCPyV-EV, and infection was determined by indirect immunofluorescence analysis of VP1 expression at 3 days post-infection. SVG-A cells were used as a positive infection control. In contrast to SVG-A cells, hCMEC/D3 cells and iPSC-EC were not infected (Fig. 1A). We next asked whether a lectin (SNA) that recognizes the sialic acid containing virus receptor would bind to either cell type. Using flow cytometric analysis, we showed that iPSC-ECs bound similar amounts of SNA to SVG-A cells (Fig. 1B), while hCMEC/D3 cells bound less SNA than either SVG-A or iPSC-ECs (Fig. 1B). Consistent with these findings, we found that hCMEC/D3 cells bound significantly less Alexa Fluor 488-labeled JCPyV than SVG-A cells or iPSC-EC cells (Fig. 1C). All three cell types, however, internalized similar amounts of labeled JCPyV (Fig. 1D). Pre-treatment of all cell types with type II neuraminidase (NA), which cleaves α2,6-linked sialic acids, significantly reduced JCPyV binding (Fig. 1E).

### iPSC-EC monolayers are stronger than hCMEC/D3 monolayers, but both cell lines form sufficiently restrictive barriers

**Fig 2:**
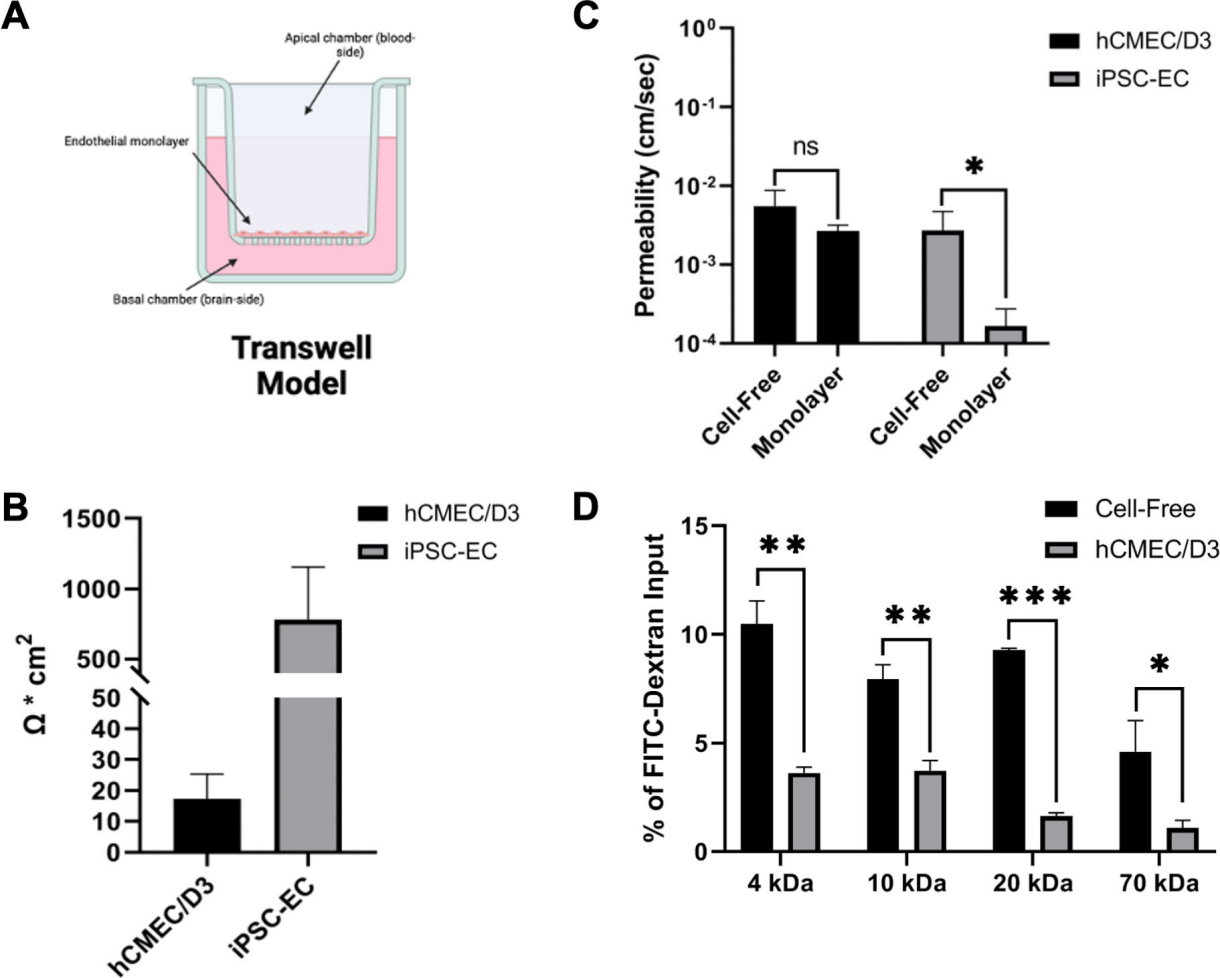
The hCMEC/D3 and iPSC-EC monolayers restrict high-molecular-weight FITC-Dextrans and sodium fluorescein. (A) Schematic of the endothelial monolayer in the transwell model. Image made using BioRender.com. (B) Transendothelial electrical resistance of hCMEC/D3 and iPSC-EC monolayers. (C) hCMEC/D3 and iPSC-EC monolayer permeability was assessed by basal accumulation of sodium fluorescein. Fluorescence of the basal supernatant was reported as the permeability in cm/s of the input sodium fluorescein added to the apical chamber. Cell-free inserts were used as a control. (D) hCMEC/D3 monolayer permeability was assessed by basal accumulation of a series of high-molecular-weight FITC-Dextrans (4, 10, 20, and 70 kDa). Fluorescence of the basal supernatant was reported as a percentage of the input FITC-Dextran solution added to the apical chamber. Cell-free inserts were used as a control. Significance was determined by a Student’s two-tailed *t* test (*P < 0.05, **P < 0.01, ***P < 0.001).

iPS cells were induced to an endothelial cell phenotype and rigorously characterized for the expression of endothelial cell markers and for polarization of the p-glycoprotein efflux transporter (Fig. S1A and B). We then modeled a BBB using cells grown on transwell culture inserts (29, 30) (Fig. 2A). hCMEC/D3 cells have been characterized to retain the properties of primary microvascular endothelial cells and are consistently used in the transwell model (32), while iPSC-ECs have been shown to form more restrictive barriers (31, 33). Consistent with prior reports (31, 32), we found that the iPSC-EC monolayer exhibited substantially greater transendothelial electrical resistance (TEER) than the hCMEC/D3 monolayer (Fig. 2B). Further demonstrating differences in barrier strength, the iPSC-EC barrier restricted passage of the low-molecular-weight sodium-fluorescein (NA-F) tracer unlike the hCMEC/D3 cells (Fig. 2C).

Studies using hCMEC/D3 monolayers commonly use high-molecular-weight dextrans to measure paracellular permeability (34, 35). Here, we found that the hCMEC/D3 monolayer semi-sequentially restricted a series of high-molecular-weight FITC-Dextrans ranging from 4 to 70 kDa, relative to cell-free inserts (Fig. 2D). hCMEC/D3 cells do not develop high TEER values in the same manner as iPSC-EC cultures; however, the use of hCMEC/D3 in transwell cultures has been well characterized as a physical and functional model of the blood-brain barrier (30, 36).

### The hCMEC/D3 monolayer restricts JCPyV passage

**Fig 3:**
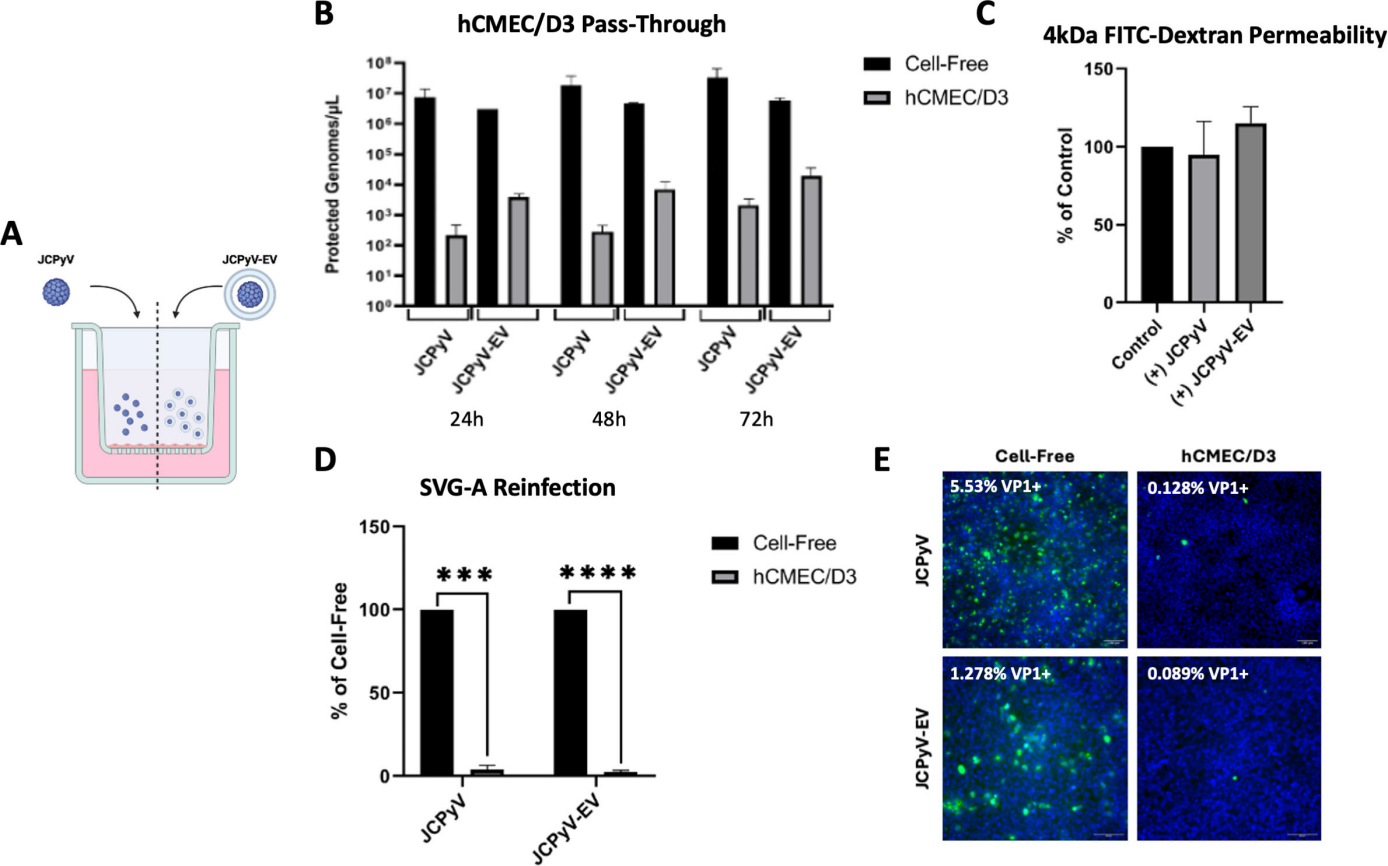
The hCMEC/D3 monolayer restricts JCPyV virions and EV-associated virus. (A) JCPyV neuroinvasion from the periphery to the brain was modeled by adding JCPyV virions or EV-associated virus (JCPyV-EV) to the apical chamber of the hCMEC/D3 monolayer and quantifying basal accumulation of virus over time. Figure made using BioRender.com. (B) Basal supernatants were collected at 24, 48, and 72 h post-virus addition to the apical chamber. Basal supernatants were treated with DNase, and the number of protected viral genomes/µL was quantified by qPCR using a VP2 primer/probe set. Significance was determined by a Student’s one-tailed *t* test. (C) hCMEC/D3 monolayer integrity following 72 h exposure to JCPyV or JCPyV-EV was evaluated by permeability to 4 kDa FITC-Dextran. Fluorescence of the basal supernatant of virus-exposed hCMEC/D3 monolayers was normalized as a percentage of the non-virus-exposed control. Significance was determined by a Student’s two-tailed *t* test. (D, E) SVG-A cells were infected with the 72 h basal supernatants from hCMEC/D3 monolayers or cell-free inserts and expanded for 9 days. (D) The magnitude of infection was reported as the % of VP1^+^ SVG-A cells. Significance was determined by a Student’s two-tailed *t* test. (E) Representative immunofluorescence images of SVG-A cells infected with basal supernatants (green = VP1, blue = cell nuclei). Asterisks were used to indicate statistical significance (***P < 0.001, ****P < 0.0001).

To model JCPyV passage across the BBB endothelium from the blood to the brain, we introduced JCPyV virions or virus-associated extracellular vesicles (JCPyV-EVs) to the apical chamber of hCMEC/D3 monolayers and measured viral accumulation in the basal chamber by quantitative PCR (qPCR) over 72 h (Fig. 3A). We found that the hCMEC/D3 monolayer significantly restricted viral accumulation in the basal chamber by several orders of magnitude at all time-points, compared to cell-free controls (Fig. 3B). To investigate whether barrier integrity was affected by persistent apical exposure to JCPyV, we measured permeability to 4 kDa FITC-Dextran and found no significant differences compared to uninfected controls (Fig. 3C). Next, we used the basal supernatants to reinfect SVG-A cells and found that they were sufficient to establish a minimal, yet detectable, infection (Fig. 3D and E). Figure S2A shows the daily TEER values associated with the hCMEM/D3 pass-through experiments in Fig. 3.

### The iPSC-EC monolayer restricts JCPyV passage

**Fig 4:**
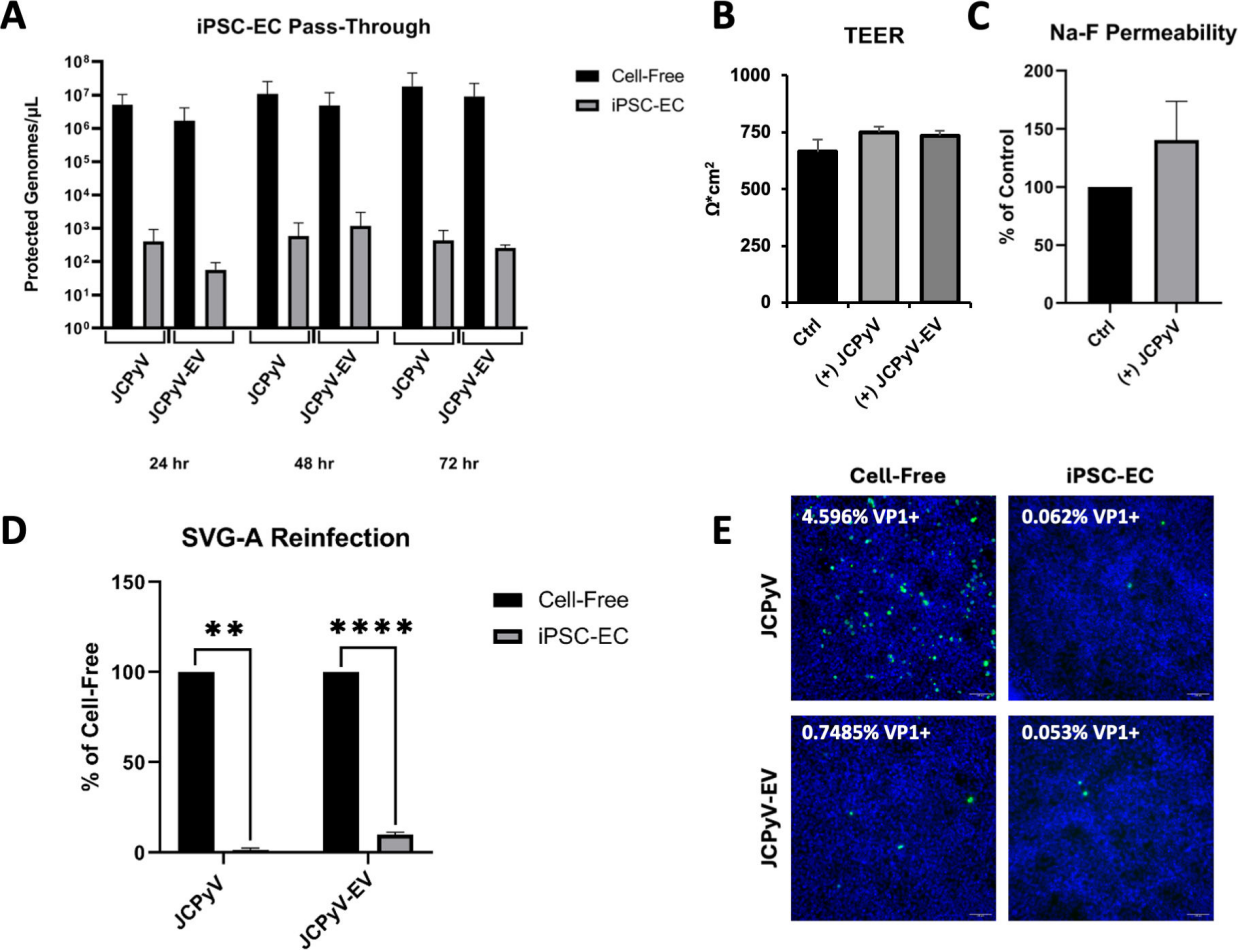
The IPSC-EC monolayer restricts JCPyV virions and EV-associated virus. (A) JCPyV neuroinvasion from the periphery to the brain was modeled by adding JCPyV virions or EV-associated virus (JCPyV-EV) to the apical chamber of the iPSC-EC monolayer and quantifying basal accumulation of virus over time. iPSC-EC monolayer integrity following 72 h exposure to JCPyV or JCPyV-EV was evaluated by TEER (B) and by permeability to sodium fluorescein (C). Fluorescence of the basal supernatant of virus-exposed iPSC-EC monolayers was normalized as a percentage of the non-virus-exposed control. Significance was determined by a Student’s two-tailed *t* test. SVG-A cells were infected with the 72 h basal supernatants from iPSC-EC monolayers or cell-free inserts and expanded for 9 days. (D) The magnitude of infection was reported as the % of VP1^+^ SVG-A cells. Significance was determined by a Student’s two-tailed *t* test. (E) Representative immunofluorescence images of SVG-A cells infected with basal supernatants (green = VP1, blue = cell nuclei). Asterisks were used to indicate statistical significance (*P < 0.05, ***P < 0.001, ****P < 0.0001).

iPSC-ECs were grown in transwell dishes for 24–48 h and then exposed to JCPyV virions or virus-associated extracellular vesicles (JCPyV-EVs). Passage of virus across the barrier was measured by quantitative PCR (qPCR) over a 72 h time course (Fig. 4A). We found that the iPSC-ECs significantly impeded viral passage from the apical to the basal chamber of the transwell culture insert, compared to cell-free controls (Fig. 4A). To investigate whether barrier integrity was affected by persistent apical exposure to JCPyV or JCPyV-EVs, we measured TEER and penetration by the small-molecular-weight tracer sodium fluorescein (Fig. 4B and C). Exposure of the cells to virus or virus-EV did not significantly alter TEER and was not sufficient to increase the penetration of the barrier by Na-F (Fig. 4C). Next, we found that the basal supernatant was sufficient to establish a minimal, yet detectable, infection in SVG-A cells (Fig. 4D and E). Figure S2B shows the daily TEER value associated with the iPSC-EC pass-through experiments in Fig. 4.

## DISCUSSION

The blood-brain barrier (BBB) is a specialized, multicellular interface that separates the brain from peripheral circulation. Consisting mainly of endothelial cells, pericytes, and astrocytic endfeet, the BBB maintains CNS homeostasis by tightly regulating the transit of macromolecules between the blood and the brain (37, 38). Different neurotropic viruses employ distinct strategies to infiltrate the brain across the BBB. For example, West Nile Virus has been shown to traverse the BBB paracellularly when endothelial barrier integrity is weakened by proinflammatory cytokines (39, 40). West Nile Virus may also employ a “Trojan Horse” strategy to enter the brain through transendothelial migration of infected monocytes (41). In contrast, the Zika Virus has been shown to cross the brain microvascular endothelium through transcytosis and productive infection of brain microvascular endothelial cells although CNS infiltration may also occur via infected monocytes (35, 42). Here, we show that the BBB endothelium is a potential site for the transcellular passage of JCPyV from peripheral circulation to the brain.

To model the brain microvascular endothelium *in vitro*, we used the SV40/hTERT immortalized endothelial cell line hCMEC/D3 as well as iPSC cells induced to an endothelial cell phenotype (iPSC-EC). Both cellular models have been used extensively to model BBB dynamics (29, 30, 34). First, we exposed both cell types to either purified virus or virus associated with EVs to determine their susceptibility to infection. Neither hCMEC/D3 cells nor iPSC-ECs were permissive to infection at 3 dpi. This is in contrast to the choroid plexus epithelial cells of the blood-cerebral spinal fluid barrier (BCSFB), which form a highly restrictive barrier to virus passage but are permissive to JCPyV infection (43, 44). However, despite the lack of productive infection, both types of neuroendothelium bound and internalized JCPyV. Binding depended on α2,6-sialic acids, as treatment with neuraminidase significantly reduced binding. These results corroborate an *in vivo* histological report from our group which showed that fluorescent JCPyV virions colocalize with the sialic acid containing attachment receptor for JCPyV, LSTc, on human brain microvascular endothelium (45).

We also found that monolayer cultures of hCMEC/D3 or iPSC-EC cells significantly restricted the apical-to-basal passage of JCPyV by several orders of magnitude but permitted a minimal amount of virus to accumulate in the basal chamber. Because we only quantified protected genomes in the basal supernatant, we can reasonably conclude that intact virions crossed the monolayers. In a reinfection assay on SVG-A cells, the hCMEC/D3 and iPSC-EC basal supernatants were infectious. That the hCMEC/D3 and iPSC-EC monolayers restricted viral passage yet permitted a small amount of JCPyV to cross suggests a “catch and release” dynamic that may occur in the physiological BBB endothelium. Brain microvascular endothelial cells might “catch” circulating JCPyV from the blood and gradually “release” infectious virions to the brain over time. Furthermore, hCMEC/D3 and iPSC-EC barrier permeability to FITC-Dextran and Na-F, respectively, were unaffected by persistent exposure to JCPyV, suggesting that the virus alone is insufficient to cause significant disruptions to barrier integrity.

We cannot exclude the possibility that JCPyV crosses the brain microvascular endothelium paracellularly when barrier integrity is disrupted. Because minimal virus was able to traverse the intact endothelial barriers, one possibility is that the majority of virus that invades the CNS to cause PML does so following heightened inflammation at the BBB (45). An inflammatory environment would impair junctional integrity, disrupt the homeostatic function of the BBB, and “open the flood-gates” to the paracellular diffusion of macromolecules between the blood and the brain (38, 46). Proinflammatory cytokines including TNFα and IFNγ have been shown to weaken endothelial barrier integrity and consequently increase West Nile Virus accumulation in the basal chamber (47). Interestingly, the barrier cells of the choroid plexus secrete pro-inflammatory chemokines following JCPyV infection (48). These chemokines, including CCL2, have been shown in studies of mouse models of inflammation to be secreted by the choroid plexus and then disrupt the neuroendothelium (49).

We should note that JCPyV quasispecies isolated from the brains and CSF of PML patients commonly exhibit VP1 point-mutations that impair LSTc binding (9–11, 50). In this report, we used the Mad1/SVE Δ (Turbo) strain of JCPyV, which does not harbor mutations that weaken viral attachment to LSTc (11, 50). Our study demonstrates that brain microvascular endothelial cells bind and internalize Turbo JCPyV virions through engagement of α2,6-linked sialic acids, but we do not explore the ability of the BBB endothelium to transport PML-VP1 mutants with deficient LSTc binding. One possibility is that JCPyV virions associate with EVs in the periphery, which enables the virus to traverse the BBB independent of LSTc. Our group has shown that EV-associated JCPyV but not purified virus can infect glial cells that lack necessary viral attachment receptors (2, 25, 26). Following this logic, peripherally derived EVs may serve as a vehicle for JCPyV neuroinvasion. A recent report found that the plasma of HIV^+^ patients who were at-risk for PML contained EVs associated with JCPyV archetype DNA (27). EV trafficking across the BBB is a dynamic and rapidly growing field of study, with several reports showing that EV can cross the barrier via transcytosis *in vitro* and *in vivo* (28). In this report, we demonstrated the ability for EV-associated virus to cross the brain microvascular endothelium and subsequently infect glial cells *in vitro*.

## MATERIALS AND METHODS

### Cells and virus

The SV40/hTERT immortalized endothelial cell line hCMEC/D3 was purchased from Creative Biolabs (NCL-2108-P020). hCMEC/D3 cells were maintained in a complete endothelial cell medium (ScienCell, cat. 1001). SVG-A glial cells were maintained in Minimum Essential Media ([MEM], Corning) supplemented with 5% fetal bovine serum (Atlanta Biologics) and 1% Antibiotic-Antimycotic (ThermoFisher). The iPSC culture IMR90-2 was purchased from WiCell and maintained on matrigel-coated plates with essential 8 (E8) medium (Thermo Fisher Scientific, 05990). Cells were passaged as needed every 4–6 days using Versene. EV-depleted medium (EVD) was used for all EV-related experiments. EVD was prepared as previously described (25, 51). 2% FBS complete endothelial cell medium was prepared and spun for at least 18 h at 100,000 × *g* in a type 45 Ti rotor (*k*-factor = 133). The supernatant was removed by pipetting, to not disturb the pellet, and was then filtered through a 0.22-µm-pore-size filter (Celltreat, Pepperell, MA). All JCPyV-EV experiments were carried out in EVD media.

The Mad-1/SVEΔ strain of JCPyV (Turbo) was prepared and purified as previously described (26, 52, 53). SVG-A cells were cultured and infected with Mad1/SVEΔ (Turbo) at an MOI of 10. Infection was allowed to spread through the culture for 3 weeks, with weekly media changes. When significant lysis and vacuolization were apparent, the infected culture was collected in whole by scraping. Cells were treated with deoxycholate, sonication, Type II neuraminidase and repeated freeze-thaw cycles to release virions from the cells. Virions were purified by ultracentrifugation through a 20% sucrose cushion, followed by cesium chloride density gradient ultracentrifugation. Purified virions were collected from the gradient using a syringe, and viral titer was quantified by qPCR comparison to a standard curve of Mad-1 plasmid DNA carried in the pBR322 vector. Primers used to quantify viral genomes are detailed in qPCR methods below. Alexa Fluor 488-conjugated JCPyV was prepared as previously described (26). Purified virions were incubated with Alexa Fluor 488 labeling dye (Thermo Fisher Scientific) for 1 h at room temperature and then dialyzed against bicarbonate buffer pH 8.3 overnight in a 10,000 kd MWCO dialysis cassette (Pierce) for 48 h, with two buffer changes.

### Differentiation of iPSCs to endothelial-like cells

iPSCs were differentiated to endothelial-like cells (iPSC-ECs), as previously described. Cells were seeded to matrigel-coated plates in E8 medium, supplemented with 10 µM ROCK inhibitor (Y-27632, R&D Systems, 1254) at a density of 12,000 cells/cm^2^. The following day, the medium was aspirated and replaced with essential 6 (E6) medium (Thermo Fisher Scientific, A1516401) to begin differentiation. E6 medium was aspirated and replaced with fresh medium every 24 h for 4 days. The medium was then changed to human endothelial serum-free medium (hESFM, Thermo Fisher Scientific, 11111) containing 10 µM all-trans retinoic acid (RA, Sigma, R2625), 10 nM fibroblast growth factor (bFGF, Peprotech, 100-18B), and 1% B27 (Thermo Fisher Scientific, 17504-044). After 48 h without media changes, the fully differentiated iPSC-ECs were dissociated with accutase and cryopreserved. Cells were resuspended in freezing medium (10% DMSO and 30% FBS in hESFM containing 10 µM RA, 10 nM bFGF, 1% B27, and 10 µM ROCK inhibitor). Cells were transferred to cryotubes at a concentration of 4–6 × 10^6^ cells/mL, placed in an isopropanol container overnight at −80°C, and then transferred to a liquid nitrogen tank for long-term storage. Cells were used within 3 months of freezing.

### Preparation and purification of EV

SVG-A cells were infected with the Mad-1/SVEΔ strain of JCPyV, MOI 10, for 2 h at 37°C, 5% CO_2_ followed by expansion for 7 days post infection in EVD media, alongside an uninfected control. The supernatant was then collected, and any debris was pelleted out at 300 × *g* in a Sorvall Legend X1R (Thermo) centrifuge for 10 min, followed by a second spin at 2000 × *g* for 10 min. The supernatant was then spun at 10,000 × *g* for 30 min using a Sorvall Lynx 6000 (Thermo), twice. Clean tubes were used for each spin step. The clarified supernatant was next transferred to Ultra Clear tubes (Beckman Coulter, Brea, CA) and spun for 70 min at 100,000 × *g* using a SW55 Ti rotor (*k*-factor = 139). The supernatant was disposed, and the pellet was resuspended in fresh filtered 1× PBS and spun for an additional 70 min at 100,000 × *g*. The pellet was resuspended in PBS-HAT in 1/200 of the original media volume and stored in liquid nitrogen. Vesicles were characterized by particle size and number using a ZetaView Quatt (Particle Metrix). The presence of virus and genome copies per particle were confirmed by qPCR using a VP2 primer/probe set as detailed in the quantitative qPCR section.

### Flow cytometry

A BD FACSCanto II (BD Biosciences) was used to perform flow cytometry experiments. A minimum of 5,000 events were collected per sample, and the median fluorescence intensity was used to calculate the magnitude of viral binding or viral internalization. Analysis was performed using FlowJo.

### Lectin binding

Cells were dissociated using Cellstripper (Corning, 25-056-CI) and resuspended in pre-chilled 1× PBS at a concentration of 5 × 10^6^ cells/mL. To label α2,6-linked sialic acid, 50 µL of the cell suspension was incubated with 40 µg/mL of FITC-conjugated *Sambucus nigra lectin* (SNA, ThermoFisher, L32479) in the dark on ice for 1 h.

### JCPyV binding

Cells were dissociated using Cellstripper (Corning, cat. 25-056-CI) and resuspended in pre-chilled PBS at a concentration of 5 × 10^4^ cells/mL. Cells were incubated with fluorescently labeled JCPyV in the dark on ice for 1 h to promote viral binding while preventing internalization. To strip α2,6-linked sialic acids prior to JCPyV binding, cells were suspended in 1× PBS adjusted to a pH of 6 and treated with 0.5 U/mL type II neuraminidase (NA, Sigma, N6514) for 1.5 h at 37°C, 5% CO_2_. Cells were briefly spun and washed with pH 7.5 PBS to inactivate residual type II NA. Cells were resuspended in pre-chilled 1× PBS and incubated with fluorescently labeled JCPyV in the dark on ice for 1 h. Following incubation, cells were washed in cold 1× PBS to remove unbound virus and resuspended in cold 1× PBS. Samples were analyzed first as-is for total fluorescent signal (internalized and externally bound JCPyV-488), immediately after which trypan blue (Corning, 25-900-CI) was added to 0.04% and samples read again. The fluorescence before quenching minus the fluorescent signal after quenching represents externally bound virus (54).

### JCPyV internalization

Cells were seeded at 5 × 10^4^ cells/well in a 24-well plate. The next day, cells were incubated with fluorescently labeled JCPyV in unsupplemented minimum essential media for 1 h at 37°C, 5% CO_2_. Cells were dissociated using 0.05% trypsin (Corning, 25,051 CI) and resuspended in pre-chilled 1× PBS. Samples were analyzed first as-is for total fluorescent signal (internalized and externally bound JCPyV-488), immediately after which trypan blue was added to 0.04% and samples read again. The fluorescent signal after trypan addition represents internalized virus, protected from quenching.

### Transwell cultures—hCMEC/D3 monolayers

hCMEC/D3 transwell cultures were established according to previously described methods (55). Transwell inserts (12 mm, 0.4 µm pore size, Corning cat. 3401) were coated with a 5:4:1 mixture of molecular biology grade water (Corning, 46-000-cl), collagen type IV (Sigma Aldrich, C5533) dissolved in 10% glacial acetic acid (Fisher Scientific, A38S-500) to 1 mg/mL, and fibronectin (Sigma Aldrich, F1141) and incubated for at least 4 h or up to overnight at 37°C, 5% CO_2_. Prior to cell seeding, the coating solution was aspirated, and inserts were dried and washed with complete endothelial medium. hCMEC/D3 cells were seeded to the apical chamber in 500 µL complete endothelial cell medium at a density of 5 × 10^4^ cells/cm^2^. 1.5 mL complete endothelial cell medium was added to the basal chamber. Cells were incubated at 37°C, 5% CO_2_ for 48 h, after which the apical and basal supernatants were aspirated and replaced with fresh complete endothelial medium. Cells were expanded for an additional 72 h, at which point all transwell experiments were conducted.

### Transwell cultures-iPSC-EC

Transwell inserts (6.6 mm, 0.4 µm pore size, Corning cat. 3378) were coated with a 5:4:1 mixture of molecular biology grade water (Corning, 46-000-cl), collagen type IV (Sigma Aldrich, C5533) dissolved in 10% glacial acetic acid (Fisher Scientific, A38S-500) to 1 mg/mL, and fibronectin (Sigma Aldrich, F1141) and incubated for at least 4 h or up to overnight at 37°C, 5% CO_2_. Prior to cell seeding, the coating solution was aspirated, and inserts were dried for 30 min at room temperature and washed with hESFM. Cryopreserved cells were thawed in a 37°C water bath, resuspended in hESFM containing 1% B27 and 10  µM ROCK inhibitor, and quantified using a hemocytometer. Cryoprotectant was removed by centrifugation for 3 min at 1,000 rpm. Cells were resuspended in hESFM and plated to transwell inserts at a density of 5 × 10^5^ cells/cm^2^. Cells were used for experiments within 24–48 h of plating.

### Permeability assays-FITC-Dextran

FITC-Dextran permeability was conducted, as previously described (55). Apical and basal supernatants were aspirated. Pre-warmed unsupplemented MEM was added to the basal chamber, and medium containing 100 µg/mL of FITC-Dextran was added to the apical chamber. Molecular weights of FITC-Dextrans are as follows: 4 kDa (Sigma, 46944), 10 kDa (ThermoFisher, D22910), 20 kDa (Sigma, FD20), and 70 kDa (Sigma, 90718). 4 kDa FITC-Dextran was used for all permeability assays following JCPyV incubation. Coated inserts lacking any cells were used as a control. Inserts were incubated for 1 h at 37°C, 5% CO_2_ in the dark, after which 150 µL of basal supernatant was collected and measured for fluorescence at 485/530 nm using a Biotek Cytation 5 Multi-Mode Plate Reader. Permeability was calculated as a percentage of the fluorescence of the FITC-Dextran input solution. As specified, permeability values were normalized to the cell-free control.

### Permeability assays—sodium fluorescein

NA-F permeability was conducted and calculated, as previously described (33). Apical and basal supernatants were aspirated. Pre-warmed unsupplemented MEM was added to the basal chamber, and medium containing 10 µM NA-F (F6377, Sigma Aldrich) was added to the apical chamber. Cells were maintained at 37°C, 5% CO_2_ in the dark. At 15, 30, 45, and 60 min, 150 µL of the basal supernatant was collected and replaced with fresh unsupplemented MEM. At 60 min, 150 µL of the apical supernatant was collected. Fluorescence of the basal and apical supernatants was measured at 485/530 nm using a Biotek Cytation 5 Multi-Mode Plate Reader, and permeability to NA-F in cm/s was calculated according to the formula described in Stebbins et al.:


$$SR,T=\left(RFU30min+\left(RFU15min*\frac{75\mu l}{750\mu l}\right)\right)$$

$$Clearance\;volume=\frac{\left(Vb*SB,T\right)}{St,60\min}$$

$$PE\;\left(\frac{cm}{\min}\right)=\left[\left(\frac{1}{1PE}\right)\right)/1000]/Area$$

### P-gp directional transport

Directional efflux of the fluorescent P-glycoprotein (P-gp) substrate rhodamine 123 was used to assess polarity of the iPSC-EC monolayer. The efflux ratio represents the difference in rhodamine 123 accumulation in the apical chamber (basal → apical transport) vs the basal chamber (apical → basal transport). A ratio greater than 2 indicates apical polarization of P-gp transporters. The efflux ratio of iPSC-EC transwell cultures was 5.5 ± 3, calculated according to the formula described in Stebbins et al.:


$$Papp,A\to B=\frac{RFU\;basal\;chamber}{RFU\;apical\;chamber*area*time}$$

$$Papp,B\to A=\frac{RFU\;apical\;chamber}{RFU\;basal\;chamber*area*time}$$

$$Efflux\;Ratio=\frac{Papp,B\to A}{Papp,A\to B}$$

### Transendothelial electrical resistance

Transendothelial electrical resistance (TEER) was measured in every experimental well using an EVOM-2 Voltohmmeter. The probe was sterilized by soaking in ethanol and equilibrated in room temperature 1× PBS before use and between each sample. Transwell resistance was read by placing one probe in the basal chamber and one probe in the apical chamber until the reading stabilized. Transwell plates were allowed to equilibrate at room temperature for 20 min prior to reading. TEER was measured daily, before any sample collections and media changes. Ω*cm^2^ was calculated by subtracting the measured value of transwell inserts without cells in media (cell free) from the measured value of the sample wells and multiplying by the transwell surface area. TEER measurements greater than 300 Ω*cm^2^ were considered adequate for barrier formation.

### JCPyV and JCPyV-EV passage across hCMEC/D3 monolayers

The hCMEC/D3 monolayer was established, as described in the section above. Medium in the basal chamber was replaced with 1.5 mL of fresh complete endothelial cell medium. Five hundred microliters of complete endothelial cell medium containing equivalent concentrations (1 ×10^8^ copies/µL genome equivalents) of protected viral genomes was added to the apical chamber, and the hCMEC/D3 monolayer was maintained at 37°C, 5% CO_2_. At each 24, 48, and 72 h post addition, 150 µL of basal supernatant was collected and replaced with fresh, pre-warmed complete endothelial cell medium (JCPyV pass through) or EVD endothelial cell medium (JCPyV-EV). Supernatants were stored at −20°C until DNase treatment and DNA extraction for qPCR assays.

### JCPyV and JCPyV-EV passage across iPSC-EC monolayers

The iPSC-EC monolayer was established, as described in the section above. Medium in the basal chamber was replaced with 1.5 mL of fresh hESFM containing 1% B27. Five hundred microliters of hESFM containing 1% B27 with equivalent concentrations of protected viral genomes (1 × 10^8^ copies/µL genome equivalents) was added to the apical chamber, and the transwell cultures were maintained at 37°C, 5% CO_2_. At each 24, 48, and 72 h post addition, 150 µL of basal supernatant was collected and replaced with fresh, pre-warmed hESFM containing 1% B27. Supernatants were stored at −20°C until DNase treatment and DNA extraction for qPCR assays.

### Genome quantification using quantitative PCR

Basal chamber supernatants were used to quantify how much JCPyV was able to breach hCMEC/D3 and iPSC-EC monolayers. To eliminate unencapsidated DNA, supernatants were pretreated with DNase 1 (M0303, New England BioLabs) for 30 min at 37°C, followed by inactivation for 10 min at 75°C. Protected DNA was extracted using the DNeasy 96 Blood & Tissue Kit (Qiagen, cat. 69581). Quantitative PCR (qPCR) was run with a Bio-Rad CFX96 detection system using the IDTFast PrimeTime qPCR Protocol with 40 cycles. The VP2 IDTFast Assay primer/probe set was used to quantify protected viral genomes/μL (probe: /5HEX/TGTTCTCCA/ZEN/CAATCTCCCAGGCTT/3IABkFQ, primer 1: CCTGGAGTGAATGCCTTTGT, primer 2: AGAGGTTAAGGCTGGCAAATC). Serial dilutions of Mad1-pBR322 plasmid were used to create a standard curve. The viral genome copy number in comparison to the standard curve was calculated using the ThermoFisher copy number tool.

### JCPyV infections

SVG-A, hCMEC/D3, or iPSC-EC cells were seeded at 5,000 cells/well in 96 well plates (Corning). The following day, cells were infected with purified JCPyV, EV-associated JCPyV, or basal supernatants from transwell viral passage experiments for 2 h at 37°C, 5% CO_2_. Following infection, the supernatant was aspirated, cells were washed with 1× phosphate-buffered saline (PBS), and 100 µL complete MEM (viral passage experiments) or EVD (for EV passage experiments) was added. Cells were maintained at 37°C, 5% CO_2_. At 3 and 6 days post infection (dpi), an additional 50 µL of complete MEM or EVD was added to each well to feed the cells. To quantify initial infection (Fig. 1), cells were fixed at 3 days post infection (dpi). To quantify viral spread (Fig. 3 and 4), cells were fixed and stained for the VP1 viral capsid protein at 9 dpi.

### Indirect immunofluorescence

At the indicated time points post infection, cells were washed with 1× PBS and fixed at −20°C with ice-cold methanol for 20 min. Following fixation, cells were rehydrated with 1× PBS and permeabilized with 0.1% Triton-X for 5 min at room temperature. Cells were incubated with the anti-VP1 antibody PAb597 diluted in 1× PBS (1:50) overnight at 4°C with gentle rocking, washed with PBS, and incubated with goat anti-mouse Alexa Fluor 488 conjugated antibody (ThermoFisher, A-11001) diluted in 1× PBS (1:1,000) for 1 h in the dark at room temperature. Cells were counterstained with 4′,6-diamidino-2-phenylindole (DAPI) diluted in 1× PBS (1:1,000) for 5 min at room temperature to label cell nuclei. A Ti2-E inverted fluorescence microscope (Nikon) was used to analyze cells at the 20× objective for VP1 staining and calculate the total cell number. The Elements High Content imaging software (Nikon) was used to perform cell count analysis. The PAb597 hybridoma was grown in-house and generously provided by Ed Harlow.

### Characterization of iPSC-ECs

Cells were plated at 5 × 10^5^ cells/cm^2^ to collagen/fibronectin-coated 96 well plates. The following day, cells were washed with 1× PBS and fixed in ice-cold methanol at −20°C for 20 min, or in cold 4% paraformaldehyde at 4°C for 20 min, according to the manufacturer’s recommendation for individual antibodies. Following fixation, cells were washed three times with 1× PBS at room temperature, followed by blocking and permeabilizing with blocking buffer (0.3% Triton-X/5% goat serum/1× PBS) at 4°C for 1 h. Cells were incubated with the relevant primary antibody overnight in the dark at 4°C with gentle rocking, after which they were washed three times with 1× PBS and incubated with the appropriate secondary antibody at 1:1,000 in 1× PBS for 1 h in the dark. Cells were washed with 1× PBS and counterstained with DAPI.

For all experiments, a Ti2-E inverted fluorescence microscope (Nikon) was used to image cells at the 20× objective. Representative immunofluorescence images were processed using ImageJ.

### Statistical analysis and graphical representation

Data represent the average of three independent replicates, and error bars represent the standard deviation. A Student’s *t* test with Welch’s Correction was used to quantify statistical significance. The use of a one- or two-tailed *t* test was specified in individual experiments. *P* < 0.05 was used as a cutoff for statistical significance, and significance was indicated by asterisks (*P < 0.05; **P < 0.01; ***P < 0.001; ****P < 0.0001). GraphPad Prism was used for statistical analysis and graphical representation. Model images were made using BioRender under the license for Brown University.

## SUPPLEMENTAL MATERIAL

Supplemental figures spectrum.00282-25-s0001.pdf Figures S1 and S2.

ASM does not own the copyrights to Supplemental Material that may be linked to, or accessed through, an article. The authors have granted ASM a non-exclusive, world-wide license to publish the Supplemental Material files. Please contact the corresponding author directly for reuse.
