# Supplementary material for: The microvascular endothelium of the blood-brain barrier is highly restrictive to JC Polyomavirus neuroinvasion
Source: Microbiol Spectr. 2025 Mar 25;13(5):e00282-25. doi: 10.1128/spectrum.00282-25 (PMC12054175; doi:10.1128/spectrum.00282-25)
Supplement: Supplemental figures — Figures S1 and S2. [file spectrum.00282-25-s0001.pdf]

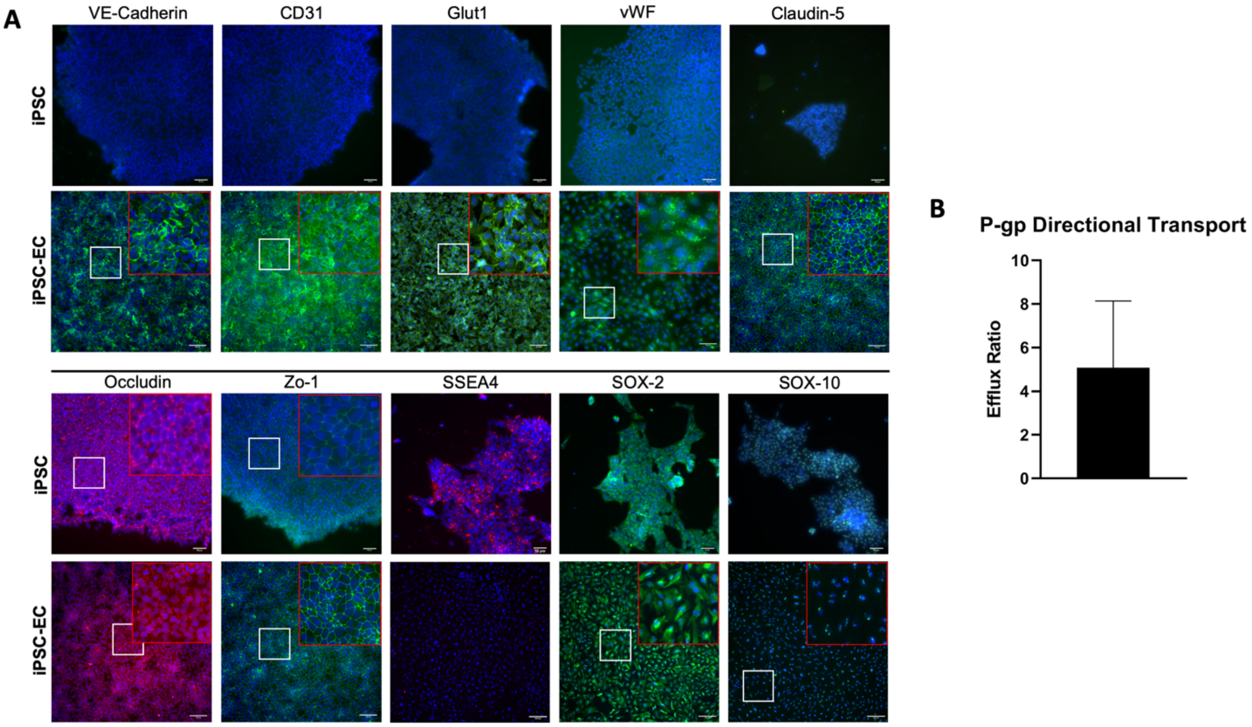

Figure S1. iPSC-ECs display characteristic properties of the BBB endothelium. A) iPSC and iPSC-ECs were labeled for phenotypic markers of the BBB endothelium and markers of stemness via indirect immunofluorescence. B) The efflux ratio of iPSC-EC transwell cultures was 5.5±3, indicating that the barrier is polarized.

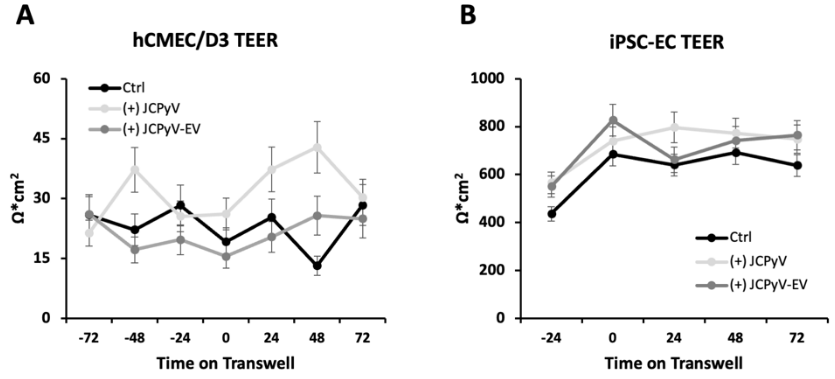

Figure S2. Transendothelial electrical resistance measurements for transwell experiments. A) Daily TEER measurements for hCMEC/D3 cultures. Time zero indicates the day of JCPyV or JCPyV-EV addition. B) Daily TEER measurements for iPSC-EC cultures. Time zero indicates the day of JCPyV or JCPyV-EV addition.
